# The Effects of Er Xian Decoction Combined with Baduanjin Exercise on Bone Mineral Density, Lower Limb Balance Function, and Mental Health in Women with Postmenopausal Osteoporosis: A Randomized Controlled Trial

**DOI:** 10.1155/2022/8602753

**Published:** 2022-06-30

**Authors:** Keqiang Li, Hongli Yu, Xiaojun Lin, Yuying Su, Lifeng Gao, Minjia Song, Hongying Fan, Daniel Krokosz, Huixin Yang, Mariusz Lipowski

**Affiliations:** ^1^Gdansk University of Physical Education and Sport, Gdańsk, Poland; ^2^School of Public Health and Management, Wenzhou Medical University, Wenzhou, China; ^3^Physical Education College, Bohai University, Jinzhou, China; ^4^Henan Province Hospital of Traditional Chinese Medicine, Zhengzhou, China; ^5^Harbin University of Physical Education, Harbin, China; ^6^School of Psychology, Beijing Sport University, Beijing, China

## Abstract

**Background:**

Postmenopausal osteoporosis (PMOP) is a common disease in older women that can severely jeopardize their health. Previous studies have demonstrated the effect of Er xian decoction (EXD) or Baduanjin exercise (BE) on PMOP. However, reports on the effect of EXD combined with BE on PMOP are limited. This study aimed to investigate the impact of EXD combined with BE on bone mineral density (BMD), lower limb balance, and mental health in women with PMOP.

**Methods:**

A 1 : 1 : 1 simple randomization technique was employed. Fifty participants with postmenopausal osteoporosis were allocated to three groups: the EXD group (EXD = 15); the BE group (BE = 18); and the combined group (EXD + BE = 17). After both 8 weeks and 16 weeks of intervention treatment, participants improved significantly with respect to BMD and the one-leg standing test (OLST), Berg balance scale (BBS), timed up and go (TUG) test, self-anxiety scale (SAS), and self-rating depression scale (SDS). The results were used to compare the effect of the intervention on BMD, lower limb balance function, and mental health in patients with PMOP.

**Results:**

Compared to the EXD and BE groups, the EXD + BE group showed the strongest effects on BMD, lower limb balance function, and mental health (*p* < 0.01). A correlation between BMD and lower limb balance and mental health was noted in the EXD + BE group. The change in mental health (SAS score) was correlated with BMD (femoral neck) improvement.

**Conclusions:**

The present study demonstrates that EXD combined with BE (EXD + BE) may have a therapeutic advantage over both monotherapies for treating BMD, lower limb balance function, and mental health in patients with PMOP. The feasibility of the approach for a large-scale RCT was also confirmed. Er xian decoction combined with Baduanjin exercise (EXD + BE) might offer a viable treatment alternative for participants with postmenopausal osteoporosis given its promising effects in disease control and treatment, with good efficacy and safety profiles.

## 1. Introduction

Osteoporosis is a systemic, multifactorial disease that causes morbidity and mortality in the elderly and is increasing in prevalence worldwide [1]. Many factors contribute to osteoporosis, such as estrogen deficiency, genetics, nutritional deficiencies, chronic diseases, and aging. Postmenopausal osteoporosis (PMOP) symptoms are mainly characterized by a decrease in bone mineral density (BMD) and changes in biochemical indicators of bone metabolism [2], which affect the stability of the lower limbs and increase the risk of fracture. Meanwhile, the decrease in BMD, bone loss, and increased fracture risk has a strong negative impact on the mental health of postmenopausal women with osteoporosis [3–8]. Western medicine is still the primary treatment for women with PMOP. However, the long-term use of Western medicine still cannot completely cure the disease. Moreover, most Western drugs are expensive, have adverse side effects, and damage the patient's body. Examples of such medications include bisphosphonates, tibolone, calcitonin, and parathyroid hormone (PTH) therapy [9–11]. Many animal studies and clinical experiments have proved that traditional Chinese medicine has a significant effect on the prevention and treatment of postmenopausal osteoporosis (PMOP) and has fewer side effects on the body than chemically synthesized medicines [12]. Therefore, the treatment of PMOP with traditional Chinese medicine will be examined in this paper.

Er xian decoction (EXD) is a multi-herb formula composed of six herbs, namely, *Rhizome curculiginis*, *Herba epimedium*, *Radix Morinda officinalis*, *Rhizome anemarrhenae*, *Cortex Phellodendron*, and *Radix Angelica sinensis.* It has several biological and pharmacological effects [13]. It has long been used to treat osteoporosis, perimenopausal syndrome, and age-related diseases in elderly patients [14–16].

EXD can improve BMD [17], promote endocrine activity and provide antioxidants [13], and treat menopause-related symptoms [18, 19]. Moreover, studies have reported that EXD is effective and safe in reducing the frequency and severity of hot flashes and improving menopausal symptoms in perimenopausal women in Hong Kong [18]. EXD showed neuroprotective effects on corticosterone-injured PC12 cells in vitro and improved depression-like behavior in mice [20]. In OVX rats, atrophy of the uterus and reduction of BMD were suppressed by treatment with EXD (*Herba epimedium*) [17]. Additionally, studies have reported that EXD can stimulate the secretion of T from Leydig cells, P from luteal cells, and E2 from granulosa cells [15].

The effectiveness of Baduanjin as an exercise intervention has been recognized in many international studies [21]. It consists of eight independent, simple, subtle, and smooth movements and is a form of qigong. BE, an important means of Chinese traditional rehabilitation therapy [21], can improve patients' blood microcirculation, transport blood calcium to the bone, promote calcium absorption, promote bone mineral salt deposition, promote and increase the proliferation and activity of bone cells, delay bone loss with age, and increase BMD [22]. Although the potential effectiveness of each movement may be different, the overall Baduanjin exercise (BE) has been demonstrated to improve physical and psychological health [23, 24]. One study has reported that a 12-week BE program significantly prevents bone loss in middle-aged women [23]. In addition, clinical observation shows that BE improves balance and fall risk in patients with senile osteoporosis [25]. Finally, a comprehensive review shows that BE facilitates improvements in psychological health and may be a suitable choice for interventions [24]. Although several studies have demonstrated a stimulatory effect of exercise on bone tissue, it is not recommended as a substitute for medical treatment.

Few published studies have investigated the effects of combination therapy using physical exercise and drugs to treat osteoporosis. Therefore, this study aims to evaluate the effect of EXD combined with BE on patients with PMOP. After 8 weeks and 16 weeks of intervention, measurements were performed to investigate the impact of the different treatments on the BMD, lower limb balance function, and mental health of study participants.

## 2. Methods and Materials

### 2.1. Study Design and Participants

#### 2.1.1. Experimental Design

**Figure 1:**
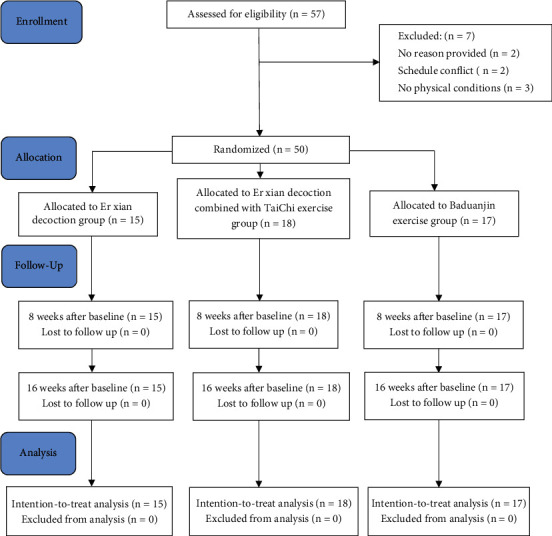
Study flow diagram of the progress through the phases of the experiment.

This study enrolled 57 participants. Seven participants dropped out after the first screening for personal reasons unrelated to the study. Two had a scheduling conflict, two did not give a reason, and three could not perform the exercises. The remaining 50 eligible subjects were enrolled and randomly assigned to the BE group (BE, *n* = 18), EXD group (EXD, *n* = 15), and combined group (BE + EXD, *n* = 17). All participants completed their intervention (Figure 1).

#### 2.1.2. Participants

From September 2021 to February 2022, 57 older citizens from six villages were randomly selected from the Lingxi Township of Wenzhou City, Zhejiang Province, China. The participants were between 50 and 70 years old, and they were required to have lived in the selected villages for more than six months in the past year. The participants completed a questionnaire survey that included the inclusion and exclusion criteria. After the screening, two participants left with no reason provided, two left the study due to scheduling conflicts, and three were excluded because their physical conditions were unsuitable. Finally, a total of 50 participants qualified for the study.

### 2.2. Diagnostic Criteria for Subject Recruitment

#### 2.2.1. Inclusion Criteria

The inclusion criteria were as follows: women are aged from 50 to 79 years old and have been in natural menopause for more than one year; the BMD T score of the lumbar spine (L2–L4) or femoral shaft is −2.5 or less; the anatomical structure of the lumbar spine is suitable for dual-energy X-ray bone density measurement, and there is no severe scoliosis, trauma, or sequelae related to bones or surgery; the participant is in good health and can move outdoors for at least 30 minutes every day; the participant can understand the research process, is willing to participate in a treatment trial, and signed the informed consent form.

#### 2.2.2. Exclusion Criteria

The exclusion criteria were as follows: the participant suffers from other severe somatic diseases or dysfunctions; the participant is unable to stand stably in place for 30 minutes; the participant has taken other anti-osteoporosis drugs (desquamate, estrogen, raloxifene) orally less than three months before entering the group; the participant suffers from mental illness or cognitive dysfunction.

### 2.3. Randomization and Allocation

Fifty participants who fulfilled the eligibility criteria were randomly allocated to three intervention-based groups: the BE group (BE, *n* = 18), EXD group (EXD, *n* = 15), or combined group (BE + EXD, *n* = 17). To ensure blinding, an independent researcher who was not a part of this study performed the randomized allocation. A 1 : 1 : 1 simple randomization technique was employed. A unique, random, computer-generated code was assigned to each participant via SPSS (version 26.0, Armonk, New York, USA). The allocation was concealed in sealed, opaque envelopes that were provided to researchers before applying the assigned interventions. All study personnel and participants were blinded to the treatment assignment for the duration of the trial. The experimental intervention times for the three groups were 8 weeks and 16 weeks.

### 2.4. Intervention

Calcium carbonate D3 tablets (Caltrate), a health supplement, can positively affect bone density. These primary drugs were given to all participants, who were asked to take two tablets once a day for 16 weeks (calcium carbonate D3 tablets (Caltrate); approval number: Sinopharm Zhunzi H10950029; manufacturer: Wyeth Pharmaceutical Co., Ltd.; specification: 600 mg).

#### 2.4.1. Baduanjin Exercise Group (BE; *n* = 17)

The participants in this group performed BE for 8 weeks and 16 weeks and continued to use their primary drugs. In the first week, they were guided by a professional Baduanjin coach. Then, they began a formal 15-week BE intervention period after mastering the moving and breathing methods. During this intervention period, the participants exercised independently. Each week, they performed the BE movements no fewer than five times, for a total duration of 45 minutes per session [26]. They prepared for exercises for 5 minutes before practice, and they practiced once a day. Follow-up was performed every two weeks by the coach who adjusted the exercise intensity according to the specific situation of the participants.

#### 2.4.2. Er Xian Decoction Group (EXD; *n* = 15)

Participants in this group continued to use their primary drugs and also took EXD. The medicinal components of EXD are as follows: *Rhizoma curculiginis* (15 g), *Herba epimedii* (15 g), *Radix Angelica sinensis* (10 g), *Cortex Phellodendri* (10 g), *Rhizoma anemarrhenae* (10 g), and *Radix Morinda officinalis* (10 g) [27]. The medicine was mixed with 800 ml water, decocted to 150 ml, and taken once daily. The participants consumed the EXD for 16 weeks [28].

#### 2.4.3. Combined Group (BE + EXD; *n* = 18)

In addition to consuming primary drugs and EXD, the participants in this group performed the BE for 8 weeks and 16 weeks. In the first week, they were guided by a professional Baduanjin coach and then began a formal 15-week intervention period of BEs after mastering the moving and breathing methods. During this intervention period, the participants exercised independently and performed the BE no fewer than five times per week for a total duration of 45 minutes per session [26]. They prepared for the exercises for 5 minutes before practice and practiced once a day. Follow-up was performed every two weeks by the coach who adjusted the exercise intensity appropriately according to the specific situation of each participant.

### 2.5. Measurements

#### 2.5.1. Bone Mineral Density (BMD)

A dual-energy X-ray absorptiometry (DEXA) scan is a valid and reliable tool for measuring BMD (Prodigy-GE Healthcare, Chicago, IL, USA) [29]. During a DEXA scan, participants lay supine on an open X-ray table. The participants were asked to keep still during the scan as the large scanning arm passed over their bodies. A trained radiologist scanned each participant's hip and spine regions for approximately 20 minutes. Using the information from the DEXA scans, the participants were classified into the normal bone mass density (score between −1 and 0 or higher), osteopenia (between −1.1 and −2.4), and osteoporosis (a score of −2.5 or less) [30]. We also calculated the *Z*-score, which compares the obtained bone density to the age-matched normal average bone and is often helpful in cases of severe osteoporosis [30, 31].

#### 2.5.2. One-Leg Standing Test (OLST)

An OLST was used to assess static balance. The participants were asked to close their eyes, stand on their preferred leg, lift the other leg to an approximately 90° angle at the knee, keep their arms by their sides, and maintain balance without using any assistive device. The test was completed when the stance foot shifted or when the lifted foot was replaced on the ground (whichever occurred first). Each participant had three attempts for each leg. The standing duration (in seconds) was recorded for each attempt, and the best (longest) score was selected for analysis [32]. Cronbach's *α* value of this test is 0.69.

#### 2.5.3. Berg Balance Scale (BBS)

The BBS includes 14 items: standing up from a sitting position; standing without support; sitting position without a backrest, but landing with both feet or putting them on a stool; sitting down from a standing position; transferring; closing eyes without support; standing with both feet together without support; stretching upper limbs and moving forward in standing position; picking up articles from the ground in a standing position; turning to look back in a standing position; turning 360 degrees; putting one foot on a step or stool in a standing position without support; and standing without support with one foot in front. The scoring standard of each item was from 0 to 4, and the total score was from 0 to 56. This range is divided into five grades: zero, poor, fair, good, and normal [33]. Cronbach's *α* value of this test is 0.77.

#### 2.5.4. Timed Up and Go (TUG) Test

In the TUG test, participants sat on a straight-back chair (the chair's seat height is about 45 cm), wearing the shoes they usually wear, and then leaned against the back of the chair with their hands crossed at their chest. After the “start” instruction, the subjects immediately stood up from the chair, walked forward for 3 meters at their normal walking gait, and turned around after passing the 3-meter marker. Then, they walked back to the chair and sat down, returning to the starting position. The participants could not receive any help during the test [34]. The time was recorded (in seconds) with a stopwatch. Before the formal test, the subjects could practice once or twice to ensure that they understood the whole test process. The formal test was conducted three times, and the average value was taken. The scoring criteria are as follows: If completion time is <10 seconds, the subject can conduct free movement. If completion time is <20 seconds, the subject can move independently. If completion time is 20–29 seconds, the subject's activity is unstable, and there is a high risk of falling. If completion time is >30 seconds, there is an obstacle to activity. Cronbach's *α* value of this test is 0.71.

#### 2.5.5. Self-Anxiety Scale (SAS)

The SAS is used to evaluate the subjective feelings of anxiety in patients and can be used as a self-assessment tool for the clinical understanding of anxiety symptoms [35]. It consists of 20 items, and the frequency of symptoms defined by the items is evaluated according to the following scales: 1–4.1 means “given the other items, ‘never or rarely' would be more natural than ‘no or few'”; 2 means “sometimes”; 3 means “most of the time”; and 4 means “always.” The scores of items 5, 9, 13, 17, and 19 must be calculated in reverse, and the rest can be calculated in sequence. The scores of the 20 items are added to get the rough score, and the rough score is multiplied by 1.25 to obtain the standard score. The critical standard of anxiety assessment in China is 50 points, and a score of 50 points or more indicates anxiety. Cronbach's *α* value of this measure is 0.62.

#### 2.5.6. Self-Rating Depression Scale (SDS)

SDS is a short-term self-rating scale compiled by Zung in 1965 [36]. It is easy to implement and can effectively reflect the symptoms of depression, together with their severity and changes. The scale consists of 20 declarative sentences and corresponding question items. Each item is equivalent to a related symptom, which is graded according to four levels: 1 is “never or rarely,” 2 is “sometimes,” 3 is “most of the time,” and 4 is “always.” Ten of the 20 items (items 2, 5, 6, 11, 12, 14, 16, 17, 18, and 20) are scored in reverse order. The scores of the 20 items are summed to obtain the rough score, and the rough score is multiplied by 1.25 to obtain the standard score. Cronbach's *α* value of this test is 0.85.

### 2.6. Sample Size

The sample size was determined based on our pilot study using G*∗*Power 3 [37]. An a priori, repeated-measure ANOVA indicated that a total sample size of 50 was needed to achieve 95% power to detect the interaction effect size of 0.21 at a 0.05 level of significance. A total sample size of 57 participants was enrolled in the study.

### 2.7. Statistical Analyses

Statistical analysis was performed using SPSS (version 26.0, Armonk, New York, USA). The Shapiro–Wilk tests were performed to determine the normality of the data distribution. Normally distributed data were expressed as means with SDs, and Student's *t*-test was used to compare between-group differences. Non-normally distributed data were presented using the median (P25, P75), and the Mann–Whitney *U* test was used. The baseline characteristics between comparison groups were analyzed using the chi-square (*χ*2) test or Fisher's exact test for categorical variables described as frequencies (percentages). We used the paired *t*-test to compare the differences within the group at baseline and at 8 and 16 weeks. An ANOVA with repeated measurements with post hoc tests was used to compare the differences between different groups, as this technique is more appropriate for examining the effect of the combined treatment on BMD, lower limb balance function, and mental health of patients with PMOP. Statistical significance was defined as *p* < 0.05, and *p* < 0.01 was the standard of high statistical significance. The correlation analysis was used to analyze the correlation between lower limb balance, changes in mental health, and BMD improvement.

## 3. Results

### 3.1. Descriptive Statistics of Sociodemographic Information of the Three Groups

The study included 50 participants with PMOP, and the largest employment group was farmers (42%). Married participants comprised 84% of the sample. No significant differences were noted among the three groups in terms of social demographic data, including age, occupation, BMI, duration of menopause, duration of PMOP, and marital status.  Table 1 shows the demographic data.

### 3.2. BMD in Three Groups at Baseline, 8 Weeks, and 16 Weeks

**Figure 2:**
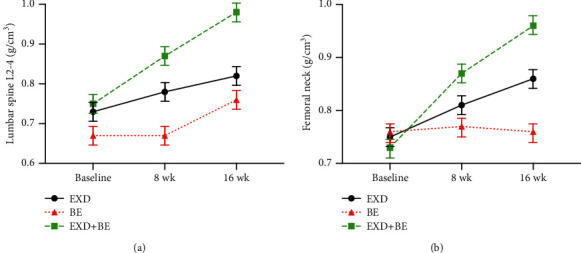
Changes in BMD in the three groups at baseline, 8 weeks, and 16 weeks.

Before the intervention, there was no significant difference between the three groups in the BMD of the lumbar spine (L2–4) and femoral neck (*p* > 0.05). After both 8 weeks and 16 weeks of intervention, the BMD of the lumbar spine and femoral neck in the EXD + BE and EXD groups was higher than that at the baseline (*p* < 0.05). Furthermore, the BMD of the lumbar spine and femoral neck was higher in the EXD + BE group than in the BE group (*p* < 0.05). The BMD of the lumbar spine and femoral neck in the EXD + BE group was higher than that in the EXD group (*p* < 0.01; Table 2 and Figure 2).

### 3.3. OLST, BBS, and TUG in Three Groups at Baseline, 8 Weeks, and 16 Weeks

**Figure 3:**
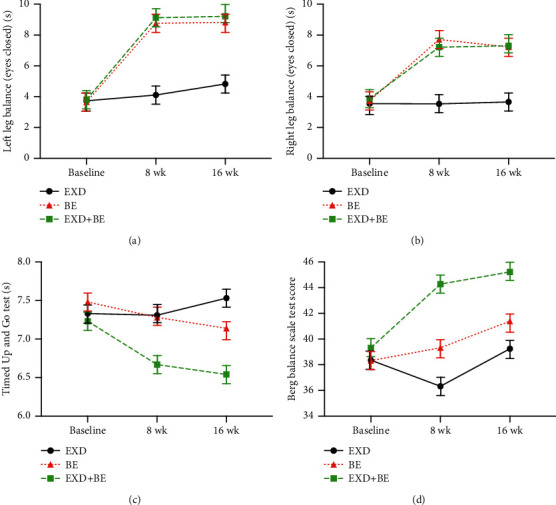
Changes in OLST, BBS, and TUG in the three groups at baseline, 8 weeks, and 16 weeks.

Before the intervention, there was no significant difference in the OLST, BBS, and TUG scores between the three groups (*p* > 0.05). After 8 weeks and 16 weeks of intervention, the OLST, BBS, and TUG scores in the EXD + BE and BE groups were higher than those at the baseline (*p* < 0.05). The OLST, BBS, and TUG scores in the EXD + BE group were higher than those in the BE group (*p* < 0.05). Meanwhile, the OLST, BBS, and TUG scores in the EXD + BE group were higher than those in the EXD group (*p* < 0.01; Table 3 and Figure 3).

### 3.4. Mental Health in Three Groups at Baseline, 8 Weeks, and 16 Weeks

**Figure 4:**
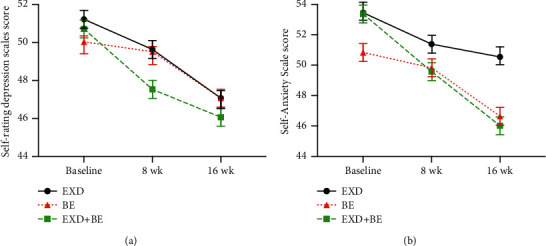
Change in mental health (SDS, SAS) in the three groups at baseline, 8 weeks, and 16 weeks.

Before the intervention, there was no significant difference between the three groups in SAS and SDS (*p* > 0.05). After 8 weeks and 16 weeks of intervention, the SAS and SDS scores in the EXD + BE, BE, and EXD groups were higher than those at the baseline (*p* < 0.05). The SAS and SDS scores in the EXD + BE group were higher than those in the BE and the EXD groups (*p* < 0.05; Table 4 and Figure 4).

### 3.5. Correlation between Changes in Mental Health, Lower Limb Balance Function, and Improvement of BMD

**Figure 5:**
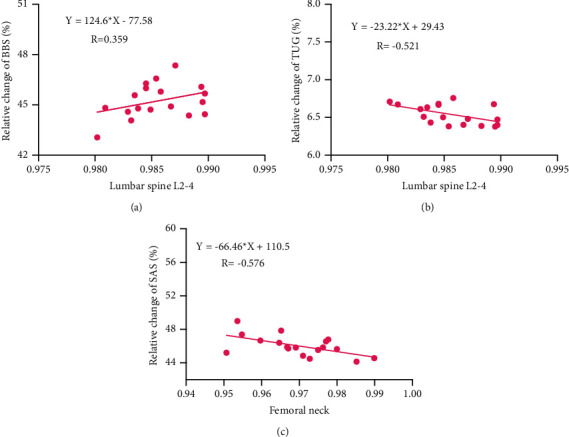
Correlations between changes in lower limb balance, mental health, and BMD.

After 8 weeks and 16 weeks of intervention, the BMD, lower limb balance, and mental health of participants in all three groups improved. We used correlation analysis to analyze the correlation between lower limb balance, changes in mental health, and BMD improvement. The results (Table 5 and Figure 5) show a significant positive correlation between lower limb balance (BBS) and BMD improvement (LS L2–4; *r* = 0.359, *p* < 0.05). Moreover, the results show a significant negative correlation between lower limb balance (TUG) and BMD improvement (LS L2–4; *r* = 0.521, *p* < 0.01). However, there is no significant correlation between BMD and OLST. In addition, the change in mental health (SAS) was negatively correlated with BMD (FN) improvement (*r* = −0.576, *p* < 0.01). However, there is no significant correlation between BMD and mental health (SDS).

## 4. Discussion

This study aimed to evaluate the effectiveness of the combination of EXD and BE on the BMD, lower limb balance function, and mental health of patients with PMOP. The research reflected the practical need to find a harmless, non-pharmaceutical intervention with minimal side effects, and the results support the feasibility and acceptability of EXD combined with BE in clinical trials. Our main finding is the significant increase of BMD in patients with PMOP in the EXD + BE group. In contrast, no significant improvement was observed in the EXD group or the BE group. Furthermore, participants in the EXD + BE group also showed a simultaneous improvement in lower limb balance function and mental health. In addition, these changes in lower limb balance function and mental health are significantly correlated with the improvement of BMD. The participants had no adverse reactions during the intervention, and all the participants were satisfied with the intervention program.

### 4.1. The Effects of EXD Combined with BE on BMD in Women with PMOP

One of our most remarkable findings is that the overall effect of the EXD + BE intervention on BMD is significantly higher than that of the BE or EXD groups. This is notable given that most studies, including both population and experimental studies, have confirmed that EXD or exercise can improve BMD [12, 14, 23, 38–42].

In addition to medication, exercise can improve BMD. Exercise has attracted much clinical attention because of its convenience, affordability, and safety, and it has been recommended by many guidelines for the prevention and treatment of osteoporosis [43, 44]. It has been proven that exercise can effectively intervene in the symptoms of PMOP [45–48].

The effect of exercise on estrogen levels may explain its therapeutic effect. Estrogen deficiency and bone resorption of osteoclasts are important causes of PMOP. Moreover, estrogen plays a very important role in the mechanism of female bone metabolism. Studies have shown that exercise can promote a slight increase in estrogen concentration [45]. Estrogen inhibits the secretion of thyroid hormone, which, in turn, reduces bone absorption, promotes the secretion of calcitonin, and reduces bone resorption. Estrogen receptors secrete factors that can effectively improve the proliferation of osteoblasts and promote bone-transforming growth factor *β*. Furthermore, the production of bone collagen molecules indirectly reduces the activity of osteoblasts, increases kidney 25-hydroxyl *α*-hydroxylase activity, and increases the production of 1, 25-(OH) 2D3 to increase the calcium absorption rate of the small intestine [48].

Meanwhile, BE can fully stretch the muscles of the spine, neck, waist, and hip; increase the flexibility of neck and waist movement and muscle strength; and stimulate the bone cells of corresponding segments to strengthen tendons and bones.

We found that combining EXD with BE (EXD + BE) had more advantageous effects on the prevention of bone loss and the improvement of BMD in patients with PMOP than either intervention on its own. However, this study shows only that exercise combined with medicine may have a therapeutic advantage over each monotherapy in improving BMD; the detailed mechanism is not completely clear as to whether this is merely an additive benefit or whether there is some synergistic effect between the two mechanisms. At present, there is no literature about the mechanism by which EXD combined with BE improves BMD. Thus, a longer-term trial would be required to evaluate the effects of EXD combined with BE on BMD.

### 4.2. The Effects of EXD Combined with BE on Lower Limb Balance Function and Mental Health in Women with PMOP

Our study also demonstrated that the participants in the EXD + BE group showed significant improvement in lower limb balance. The present study supports previous studies showing the improvement of lower limb balance function from EXD and BE [17, 21, 40, 49–51]. In addition, there is a significant positive correlation between lower limb balance (BBS test score) and BMD improvement (lumbar spine L2–4; *r* = 0.402, *p* < 0.05). Meanwhile, we observed a significant negative correlation between lower limb balance (TUG test score) and BMD improvement (femoral neck; *r* = 0.661, *p* < 0.01). However, there was no significant correlation between BMD and lower limb balance (OLST). These findings were consistent with results reported in other studies [52, 53].

Second, we observed significant improvements in mental health from EXD combined with BE. These improvements are related to improving BMD in patients with PMOP, and this result is consistent with the research of other scholars [13, 54–59]. In addition, the change in mental health (self-anxiety score) was negatively correlated with BMD improvement (lumbar spine L2–4; *r* = −0.625, *p* < 0.01). However, we observed no significant correlation between BMD and mental health (self-rating depression score). These findings are in line with other studies [60].

In summary, positive effects of EXD combined with BE were observed on lower limb balance and mental health in women with PMOP, but the detailed mechanisms need further study.

## 5. Limitations of the Study

This study has several limitations. First, the study has a small sample size for exercise intervention research. As a result, its reference value for new clinical intervention methods is limited.

Second, our study did not add clinical data for auxiliary measurement, which led to an increase in the deviation of the research results. The patients with PMOP in the EXD + BE group experienced interference and influence on indexes related to the investigation, especially on mental health. This is due to an increase in group communication and the Hawthorne effect.

Finally, as this study lacks a follow-up process after the intervention, it cannot determine whether EXD combined with BE will maintain the influence on BMD in the long term. At present, there is no more intensive study on the combination of EXD and BE, and the mechanism of the influence remains unclear.

## 6. Conclusion

The 16-week intervention of EXD combined with BE improved the BMD of patients with PMOP, especially the density of the lumbar spine (L2–4) and femoral neck. We also found that EXD combined with BE can improve balance and mental health in patients with PMOP. In addition, our study shows that BE is an effective, safe, and helpful exercise that can improve the physical and mental health of women with PMOP. In the future, research should focus on interventions involving combinations of non-pharmaceutical treatments or the lowest dose of drugs that can provide health benefits to different groups.

## Figures and Tables

**Table 1:** Demographic data.

Variable		EXD (*n* = 15)	BE (*n* = 17)	EXD + BE (*n* = 18)	*p*
Age (years)		56.41 ± 1.68	57.02 ± 1.64	57.31 ± 1.48	0.317
Employment status	Worker	4 (26.6%)	5 (29.4%)	5 (27.8%)	0.578
	Manager	0	2 (11.8%)	0	
	Farmer	6 (40%)	7 (41.2%)	8 (44.4%)	
	Other	5 (33.4%)	3 (17.6%)	5 (27.8%)	
BMI (kg/m^2^)		24.36 ± 2.03	24.48 ± 2.09	24.54 ± 1.99	0.486
Duration of menopause (years)		6.44 ± 1.81	6.51 ± 1.23	6.23 ± 2.21	0.37
Duration of PMOP (years)		4.66 ± 1.61	4.47 ± 1.24	4.31 ± 1.43	0.547
Marital status	Married	13 (86.7%)	14 (82.4%)	15 (83.3%)	0.942
	Other	2 (13.3%)	3 (17.6%)	3 (16.7%)	

**Table 2:** Changes in BMD in the three groups at baseline, 8 weeks, and 16 weeks (g/cm^3^, *x* ± *s*), *n* = 50.

Variable by group	Mean (SE)				From baseline to 16 weeks, mean (95% CI)	
	No.	Baseline	8 wk	16 wk	Within-group change	Between-group difference change
Lumbar spine L2−4 (g/cm^3^)						
EXD	15	0.73 ± 0.01	0.78 ± 0.03	0.82 ± 0.02	0.09 (0.07 to 0.1)^a^	NA
BE	17	0.72 ± 0.02	0.67 ± 0.01	0.76 ± 0.01	0.04 (0.03 to 0.05)	NA
EXD + BE	18	0.75 ± 0.01	0.87 ± 0.01	0.98 ± 0.01	0.23 (0.22 to 0.24)^a^	NA
EXD vs. BE	NA	NA	NA	NA	NA	0.06 (0.05 to 0.07)
EXD vs. EXD + BE	NA	NA	NA	NA	NA	−0.15 (−0.16 to −0.14)^a^
BE vs. EXD + BE	NA	NA	NA	NA	NA	−0.22 (−0.23 to −0.21)^a^
Femoral neck (g/cm^3^)						
EXD	15	0.75 ± 0.01	0.81 ± 0.01	0.85 ± 0.01	0.1 (0.09 to 0.11)^a^	NA
BE	17	0.75 ± 0.01	0.77 ± 0.01	0.75 ± 0.01	0 (−0.01 to 0)	NA
EXD + BE	18	0.73 ± 0.01	0.87 ± 0.01	0.97 ± 0.01	0.24 (0.23 to 0.25)^b^	NA
EXD vs. BE	NA	NA	NA	NA	NA	0.1 (−0.09 to 0.11)^a^
EXD vs. EXD + BE	NA	NA	NA	NA	NA	−0.11 (−0.12 to −0.1)^a^
BE vs. EXD + BE	NA	NA	NA	NA	NA	−0.21 (−0.22 to −0.2)^a^

*Note.* BE: BE group; EXD: EXD group; EXD + BE: EXD combined with BE; LS: lumbar spine L2–4; FN: femoral neck; NA: not applicable. a: *p* < 0.05, b: *p* < 0.01.

**Table 3:** Changes in OLST, BBS, and TUG in the three groups at baseline, 8 weeks, and 16 weeks (*x* ± *s*), *n* = 50.

Variable by group	Mean (SE)				From baseline to 16 wk, mean (95% CI)	
	No.	Baseline	8 wk	16 wk	Within-group change	Between-group difference change
Left leg balance (eyes closed), s						
EXD	15	3.74 ± 0.17	4.11 ± 0.15	4.83 ± 0.18	1.09 (0.956 to 1.22)^a^	NA
BE	17	3.66 ± 0.22	8.76 ± 0.25	8.82 ± 0.18	5.16 (5 to 5.31)^b^	NA
EXD + BE	18	3.81 ± 0.17	9.12 ± 0.13	9.21 ± 0.17	5.4 (5.26 to 5.53)^b^	NA
EXD vs. BE	NA	NA	NA	NA	NA	−3.98 (−4.11 to −3.85)^b^
EXD vs. EXD + BE	NA	NA	NA	NA	NA	−4.38 (−4.51 to −4.25)^b^
BE vs. EXD + BE	NA	NA	NA	NA	NA	−0.39 (−0.52 to −0.27)
Right leg balance (eyes closed), s						
EXD	15	3.55 ± 0.07	3.54 ± 0.06	3.66 ± 0.19	0.11 (−0.02 to 0.24)	NA
BE	17	3.74 ± 0.15	7.71 ± 0.18	7.23 ± 0.18	3.48 (3.36 to 3.61)^b^	NA
EXD + BE	18	3.88 ± 0.22	7.21 ± 0.18	7.30 ± 0.19	3.41 (3.26 to 3.57)^b^	NA
EXD vs. BE	NA	NA	NA	NA	NA	−3.56 (−3.7 to −3.42)^b^
EXD vs. EXD + BE	NA	NA	NA	NA	NA	−3.63 (−3.77 to −3.5)^b^
BE vs. EXD + BE	NA	NA	NA	NA	NA	−0.07 (−0.2 to 0.05)
TUG, s						
EXD	15	7.33 ± 0.18	7.31 ± 0.16	7.53 ± 0.24	0.19 (−0.01 to 0.38)	NA
BE	17	7.48 ± 0.23	7.28 ± 0.17	7.14 ± 0.18	−0.34 (−0.49 to −0.19)^a^	NA
EXD + BE	18	7.23 ± 0.14	6.67 ± 0.16	6.54 ± 0.19	−0.68 (−0.77 to −0.59)^b^	NA
EXD vs. BE	NA	NA	NA	NA	NA	0.38 (0.24 to 0.52)
EXD vs. EXD + BE	NA	NA	NA	NA	NA	0.98 (0.85 to 1.12)^c^
BE vs. EXD + BE	NA	NA	NA	NA	NA	0.6 (0.47 to 0.73)^b^
BBS						
EXD	15	38.36 ± 2.08	36.33 ± 1.15	39.24 ± 1.38	0.87 (−0.43 to 2.18)	NA
BE	17	38.33 ± 1.57	39.33 ± 1.78	41.39 ± 2.16	3.06 (1.78 to 4.33)^a^	NA
EXD + BE	18	39.31 ± 1.26	44.27 ± 1.58	45.23 ± 1.02	5.92 (5.12 to 6.72)^b^	NA
EXD vs. BE	NA	NA	NA	NA	NA	−2.15 (−3.29 to −1.01)^a^
EXD vs. EXD + BE	NA	NA	NA	NA	NA	−5.99 (−7.11 to −4.86)^c^
BE vs. EXD + BE	NA	NA	NA	NA	NA	−3.84 (−4.92 to −2.75)^b^

*Note.* BE: BE group; EXD: EXD group; EXD + BE: EXD combined with BE; OLST: one-leg standing test; BBS: Berg balance scale test score; TUG: timed up and go test; NA: not applicable. a: *p* < 0.05, b: *p* < 0.01, c: *p* < 0.001.

**Table 4:** Change in mental health (SDS, SAS) in the three groups at baseline, 8 weeks, and 16 weeks (*n* = 50).

Variable by group	Mean (SE)				From baseline to 16 weeks, mean (95% CI)	
	No.	Baseline	8 weeks	16 weeks	Within-group change	Between-group difference change
SDS						
EXD	15	51.22 ± 1.74	49.63 ± 1.72	47.07 ± 2.19	−4.14 (−5.33 to −2.95)^b^	NA
BE	17	50.03 ± 1.85	49.51 ± 1.38	47.06 ± 1.81	−2.96 (−4.45 to −1.47)^a^	NA
EXD + BE	18	50.71 ± 2.04	47.53 ± 2.01	46.06 ± 2.26	−4.64 (−6.13 to −3.16)^b^	NA
EXD vs. BE	NA	NA	NA	NA	NA	0.01 (−1.48 to 1.5)
EXD vs. EXD + BE	NA	NA	NA	NA	NA	1.01 (−0.46 to 2.49)^a^
BE vs. EXD + BE	NA	NA	NA	NA	NA	1 (−0.42 to 2.43)^a^
SAS						
EXD	15	53.45 ± 1.94	51.39 ± 2.11	50.55 ± 2.06	−2.89 (−4.67 to −1.12)^a^	NA
BE	17	50.84 ± 1.83	49.83 ± 1.82	46.65 ± 2.28	−4.18 (−5.38 to −2.98)^b^	NA
EXD + BE	18	53.37 ± 1.83	49.58 ± 1.31	46.02 ± 1.23	−7.34 (−8.56 to −6.13)^c^	NA
EXD vs. BE	NA	NA	NA	NA	NA	3.89 (2.54 to 5.24)^b^
EXD vs. EXD + BE	NA	NA	NA	NA	NA	4.52 (3.19 to 5.86)^b^
BE vs. EXD + BE	NA	NA	NA	NA	NA	0.63 (−0.65 to 1.92)

*Note.* BE: BE group; EXD: EXD group; EXD + BE: EXD combined with BE; SAS: self-anxiety scale; SDS: self-rating depression scales; NA: not applicable. a: *p* < 0.05, b: *p* < 0.01, c: *p* < 0.001.

**Table 5:** Correlations between changes in lower limb balance, mental health, and BMD.

Variable	BBS	TUG	OLST	SAS	SDS
LS L2−4	0.359^a^	−0.521^b^	−0.041	0.353	0.063
FN	−0.089	0.096	0.116	−0.576^b^	−0.266

*Note.* The data in the table are the correlation coefficient (*r*). a: *p* < 0.05; b: *p* < 0.01. LSL2–4: lumbar spine L2–4; FN: femoral neck; OLST: one-leg standing test; BBS: Berg balance scale test score; TUG: timed up and go; SAS: self-anxiety scale; SDS: self-rating depression scales.

## Data Availability

The data used to support the findings of this study are available from the corresponding author upon request.
